# A Review of Temporary Permanent Pacemakers and a Comparison with Conventional Temporary Pacemakers

**DOI:** 10.19102/icrm.2019.100506

**Published:** 2019-05-15

**Authors:** Keith Suarez, Javier E. Banchs

**Affiliations:** ^1^Section of Electrophysiology & Pacing, Division of Cardiology, Department of Medicine, Baylor Scott & White Temple Memorial Hospital, Baylor Scott & White Health, Dallas, TX, USA

**Keywords:** Active fixation lead, cardiac pacing, pacemaker, passive fixation lead

## Abstract

Temporary cardiac pacing is commonly used in patients with life-threatening bradycardia and serves as a bridge to implantation of a permanent pacemaker (PPM). For years, passive fixation leads have been used for this purpose, offering the advantage of that they can be placed at bedside. The downside, however, is that patients must remain on telemetry and bed rest until lead removal due to the risk of displacement and failure to capture. Even then, the latter cannot always be prevented. Temporary cardiac pacing with passive fixation leads has also been related to a higher incidence of infection and venous thrombosis, delayed recovery, and increased length of stay. Thus, over the last couple of decades, pacemaker leads with an active fixation mechanism have become increasingly used. This is known as a temporary PPM (TPPM) approach, which carries a very low risk of lead dislodgement and allows patients to ambulate, among other advantages. Here, we performed a review of the literature on the use of TPPMs and their advantages over temporary pacemakers with passive fixation leads and in order to evaluate the advantages and disadvantages of active and passive fixation leads in temporary cardiac pacing. Most articles found were case reports and case series, with few prospective studies. We excluded documents such as editorials and image case reports that provided little to no useful information for the final analysis. The literature search was performed in PubMed, Google Scholar, and other databases and articles written in English and Spanish were considered. Articles were screened up to January 2017. The search keywords used were “temporary permanent pacemaker,” “external permanent pacemaker,” “active fixation lead,” “explantable pacemaker,” “hybrid pacing,” “temporary permanent generator,” “prolonged temporary transvenous pacing,” and “semipermanent pacemaker.” A total of 24 studies with 770 patients were ultimately included in our review. The age group was primarily above the sixth decade of life, with the exception of one that included pediatric patients. Indications for pacing included device infection, sick sinus syndrome, atrioventricular block, ventricular tachycardia, and bradyarrhythmias associated with systemic illness. The duration of TPPM usage varied from a few days up to 336 days. A total of 18 (2.3%) TPPM-related infections were reported, in which the duration of TPPM use was less than 30 days in at least 15 patients. Loss of capture was documented in only eight patients (1.0%). Complication rates varied from 0% to 30%, with the highest event rates being present in studies that used femoral venous access. In conclusion, although no high-quality studies were identified in our literature search, we found the data retrieved suggest the association of overall favorable outcomes with the use of TPPMs. Device placement and removal typically involve a simple procedure, although fluoroscopy, usually applied in the cardiac catheterization laboratory, is necessary for implantation, which could represent an additional risk in a patient who is already hemodynamically unstable. When possible, a screw-in-lead pacemaker should be used for temporary pacing.

## Introduction

Initial descriptions of pulsed electrical stimulation to the heart can be attributed to J. A. McWilliam in the late 19th century.^1^ Subsequently, the first pacemaker device was built by the American physiologist Albert Hyman in 1932. In 1952, Drs. John Callaghan and Wilfred Bigelow and engineer Jack Hopps developed a bipolar catheter able to provide endocardial stimulation. Zoll Medical Corporation (Chelmsford, MA, USA) later developed an external pacing system with cutaneous electrodes. In 1959, Seymour Furman and John Schwedel were able to provide endocardial stimulation by utilizing a lead inserted through the internal jugular vein. The first attempts to employ an implantable pacemaker were performed in Sweden in 1958.^1^ Most publications only refer to Furman when addressing the history of pacemakers.

Pacemakers function by way of electrically stimulating the myocardium to increase the heart rate for the treatment of bradyarrhythmias, or, in specific cases, to prevent or treat a tachyarrhythmia (eg, QT-shortening in long QT syndrome, circuit entraining in atrial flutter and ventricular tachycardia).^2,3^ Their use can be either temporary or permanent, depending on the indication. Temporary pacing is preferred in the setting of an emergency, since it is more readily available. Temporary pacing can serve as a bridge to a permanent device or recovery, although the time to recovery can be lengthy in conditions such as Guillain–Barré disease, Lyme disease, and tetanus.^4,5^

The placement of both permanent pacemakers (PPMs) and implantable cardioverter-defibrillators in the United States increased from 1997 to 2004 by 19% and 60%, respectively.^6^ Most patients who receive these devices are elderly and, as this age group continues to grow, the number of devices implanted will likely increase—as will the rate of complications. An analysis from 1997 to 2004 in the United States population reported that 70% of patients who received a device were older than 65 years of age.^7^

Patients with a PPM who develop a pocket infection, secondary bacteremia, or endocarditis have a class I indication for complete removal of the device due to the high recurrence of infection associated with antibiotic therapy only.^7,8^ However, if the patient happens to be pacemaker-dependent, they would require temporary pacing in such a situation until the infection has been treated. Prior studies have suggested that the incidence of cardiac implantable electronic device (CIED) infections is 1% to 7%, with a 2.8-fold increase for PPMs and a six-fold increase for ICDs occurring between 1996 and 2003.^7^

**Figure 1:**
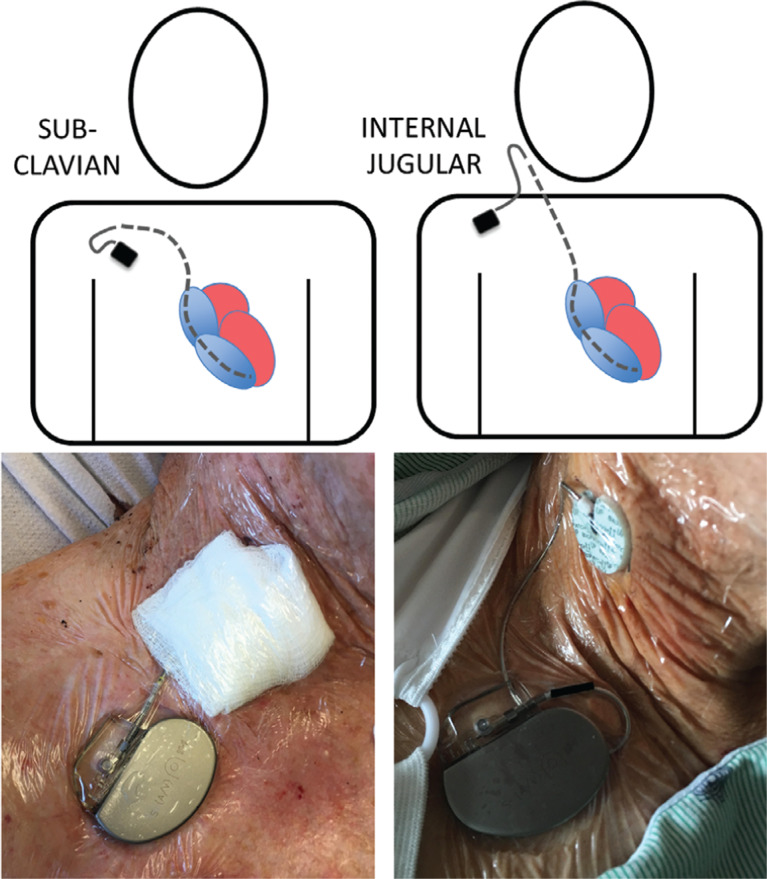
Example of a TPPM. Top: Diagrams of the lead (dotted line) placed via the subclavian (left) and internal jugular (right) approaches. Bottom: External pacemaker generator taped to the skin in each instance.

The leads more commonly used for temporary pacing are leads with no or passive fixation. Some have tines at the distal end and are positioned so that they can hold onto myocardial trabeculations. This feature heightens the risk of lead dislodgement when compared with the composition of an active fixation lead, which is also known as a temporary PPM (TPPM) lead **(Figure 1)**.^9^ Some risk factors for dislodgement are modifiable (eg, noncooperative patient,^10^ inadvertent movement of the limbs, site of venous access, inadequate positioning of the lead), while others are more difficult to troubleshoot (eg, ventricular contraction, anatomy of the right heart and great veins, nonfixation nature of the lead). The reported incidence of dislodgement varies among publications (10%–60%) and is consistently higher with passive fixation leads.

In a review article, the most common complications reported with passive fixation leads were failure of venous access (15%), failure to place a lead (10%), and sepsis (9%).^11^ Hyman et al. studied 1,022 patients at the Mayo Clinic who required conventional temporary pacing.^9^ Lead dislodgement occurred in 17.9% of patients and was the most common complication observed. The overall mortality rate was reported to be 17.6% and it was not clear as to whether or not this was a consequence of the temporary pacing itself or other factors. Another single-center retrospective study with 530 cases described a dislodgement rate of 9%, with 99% of venous access occurring through the femoral route.^10^ A total of 34 patients died, with three deaths being attributed to complications associated with the pacemaker (0.6% of all cases; 8.8% of all deaths).

The occurrence of deep vein thrombosis (DVT) and pulmonary embolism correlates primarily with the route of venous access. Nolewajka et al. studied venograms and autopsies that were completed in patients with femoral venous pacemakers.^12,13^ The incidences of femoral DVT and pulmonary embolism were 34% and 50%, respectively. Some physicians anticoagulate all of their patients, which thus adds bleeding as a potential other complication.^10^ Interestingly, a separate report of 113 patients with temporary pacemakers showed that only femoral pacemakers caused pulmonary embolism as compared with brachial ones.^14^ This route has become much less popular over time, and a shift toward utilizing the right internal jugular vein route instead was even highlighted at the time of Hyman et al.’s study.^9^ Local infection and sepsis are also known to occur more frequently in conjunction with femoral venous access.^14^

Other strategies were considered in previous decades before active fixation leads came into play. Most of these were applied in patients with a history of device infection who required temporary pacing during antibiotic treatment. In a study from 1971, four patients with infected devices were managed by opening the pocket, performing debridement, and reclosing the pocket right after.^15^ In 1984, investigators evaluated six patients who presented with pacemaker erosion.^16^ They were managed by way of exteriorizing the device and attaching it instead to the patient’s neck. Once antibiotics were completed, the infected device was replaced by a new one. Another case series studied a similar protocol and reported good outcomes as well.^17^ One study did reveal a higher recurrence rate of infection of 77% if only the generator was removed versus a rate of 8% if the leads were extracted too.^18^ The use of antibiotic therapy added to wound care with no device removal resulted in poor infection resolution, constituting the reason for why this approach is not recommend at the present time.^19^

In 1973, researchers employed a pacing method known as semipermanent pacing.^20^ In this approach, they placed a lead through the cephalic vein and connected it to a temporary pacemaker. If, after a variable period of time, this lead remained in a stable position, it was then connected to a PPM. In 1984, the use of external PPMs in DDD mode for temporary pacing was reported in 13 patients for the treatment of bradyarrhythmias and overdrive pacing.^21^ Eight patients benefited from treatment with and nine were ambulatory while using this device. Other authors have reproduced these findings.^22^ In postcardiac surgery patients, epicardial leads can be connected to an exteriorized extension and a temporary pacemaker. These leads can be later used for permanent pacing if necessary.^23^ Furthermore, in addition, epicardial leads are located outside of the intravascular space and have a lower risk of bloodstream infections. One study found TPPM patients to have a longer hospital stay than those with epicardial leads, although the reason for this finding was not clear.^24^

## Methods

### Objectives

In this study, we aimed to determine the advantages and disadvantages of employing TPPMs with active fixation leads versus standard temporary pacing. Specifically, we evaluated the length of hospital stay in terms of number of days, rate of secondary infections and venous thrombosis, incidence of loss of capture, overall rate of complications, costs, and deaths.

### Search strategy

An online search of the PubMed, Google Scholar, OVID, and EBSCO databases was performed. We searched for articles written in either English and/or Spanish and identified all relevant articles available until January 2017. The search words applied were “temporary permanent pacemaker,” “external permanent pacemaker*,” “*active fixation,” “explantable pacemaker*,” “*hybrid pacing,” “temporary permanent generator,” “prolonged temporary transvenous pacing,” and *“*semipermanent pacemaker.”

No systematic reviews, meta-analyses, or randomized control trials were found. Most articles included were full-text versions and included case reports, case series, and prospective observational studies. We excluded articles with insufficient information available as well as review articles. If an abstract was deemed to have sufficient information, it was included. One study evaluating the new Tempo Lead (BioTrace Medical, San Carlo, CA, USA) presented at the 2016 Transcatheter Cardiovascular Therapeutics meeting was also excluded.^25^

Variables included in our analysis were age, number of patients, follow-up time, duration of temporary pacing, single-group versus comparison-group study, rate of secondary infections, rate of lead dislodgement, single-chamber versus dual-chamber pacing, pacing threshold, death, average time to discharge from implantation of the temporary lead, costs, overall complications, and early ambulation. Relevant data were extracted from the articles and then represented in an Excel spreadsheet (Microsoft Corp., Redmond, WA, USA) to later generate tables **(Tables 1–4)**. Information on certain variables was missing in some studies.

## Results

Thirty-one relevant articles were found. Of these, seven were excluded because they were editorials, review articles, or had insufficient information. This left us with 24 articles. Six studies did not have a clear design method; a total of five were case reports; and, among the case series identified, three were prospective, seven were retrospective, and one combined a retrospective control group with a validation prospective group. The prospective studies were not randomized. Four studies reported having conflicts of interest and another four stated having none.

Martin et al. appeared to be the first to publish a report on the use of TPPMs.^26^ Their publication was available as a supplement. No lead dislodgements were reported, and patients were able to ambulate quickly without a need for telemetry. Two deaths occurred, although neither happened as a complication of the pacemaker implant.

All studies were single-center. Eight reported the use of atrial pacing with active fixation leads. Limited data were available about the use of temporary dual-chamber pacing and tunneled leads. One study was not clear regarding the duration of temporary pacing. Most used the internal jugular vein for access and, as second option, the subclavian vein. Seven studies reported on ambulation, while only two quantified the number of patients who did ambulate. Other studies only mentioned whether patients were allowed to ambulate or not. Regarding complications, only one publication did not report on the rate of TPPM infection, while two did not report on loss of capture. Little was reported on secondary deep venous thrombosis. The overall complication rate (excluding death) ranged between 0% and 30%. No complications occurred in 12 studies, while seven studies reported the rate of complications to be between 3% and 10%. Pecha et al. reported no complications after a mean follow-up time of 21.2 months including recurrent infection, lead dislodgement, or death.^27^ Zei et al. reported a case series of 62 patients with no documentation of lead dislodgements, device infections, or perforations after a median duration of temporary pacing for 7.5 days.^28^ Most complications were observed in three studies in which only femoral access was used; De Cock et al. found rates of 26% and 30%, respectively,^29,30^ while Garcia et al. noted a rate of 17%.^31^

Among the 24 articles, a total of 770 patients were studied. Most patients were of an advanced age. The study from Pinto et al. was the only one that included pediatric patients.^32^ Eighteen studies reported on gender distribution, with a total of 253 males (64.9%) and 137 females (35.1%) having a TPPM placed. Indications for the use of TPPM included device infection, bradyarrhythmias, ventricular tachycardia, and transcatheter aortic valve replacement **(Table 1)**. Device infection was cited as the most common indication. Kornberger et al. reported TPPM use for this indication in 70% of their patients, while, in Rastan et al.’s study, such was the indication in all of 10 patients.^33,34^ When reported, the duration of TPPM was widely variable and most often ranged between 10 days and 20 days. The lengthiest duration of TPPM was 36 months, as reported by Pecha et al.,^27^ while the shortest was one day, per Braun et al.^35^ **(Table 2)**.

Three studies had a control group with passive fixation leads, and one study compared TPPMs with epicardial leads. After excluding patients in the control groups who were treated with passive fixation, the total number of patients with TPPMs was 708. We then calculated the total percentage of patients with TPPMs who developed an infection to be 2.5%. For loss of capture, we found eight patients in the TPPM group were affected, which corresponds to 1.7% of the total number of patients **(Table 3)**. Among individual studies, we highlight De Cock, who demonstrated a lead dislocation of 5% in TPPM patients versus that of 33% in passive lead pacing patients and total adverse events rates of 30.6% and 58.1%, respectively.^29^ This difference was evident after 5.8 days ± 2.9 days of follow-up.^29^ Chihrin et al. only reported one dislodgement out of 20 patients.^36^ Amraoui et al. saw no dislodgements in 80 patients treated with TPPM placement.^24^

Of the 24 articles reviewed, a total of 18 infections of the TPPM system were reported **(Table 3)**. This number could have been even smaller if the venous access in De Cock et al.’s studies would have been subclavian or jugular rather than femoral.^24,28^ Furthermore, these two investigations reported 11 of the 18 infections that we identified in our literature search. In Kornberger et al.’s study, three TPPMs were removed due to signs of systemic infection, although it was never proven that TPPM usage was the culprit.^37^ In Kawata et al.’s study, the only patient known to have a complication had a lead vegetation and their lead was replaced.^38^

Thirteen studies reported pacing thresholds. All were measured below 1.5 V except in a study by De Cock et al. that reported a range of 1.36 V ± 0.65 V.^30^ One study reported a lower pacing threshold in the conventional pacing group, although the difference did not appear to be clinically significant.^29^ It improved in the TPPM group after a 24-hour period. Braun et al. reported a median pacing threshold of 0.6 V in the active fixation lead group, which was minimally lower when compared to that in the passive lead group.^35^ Additionally, six studies reported on the average time to discharge. The mean time varied from 11.3 days to 30.7 days. Early discharge was more likely to be achieved in patients with less severe device infections and bradyarrhythmias.

All studies reported a death rate **(Table 3)**. Specifically, there were 84 deaths reported, but only six of these were deemed by the authors to be attributed in some fashion to the pacemaker itself. Most of the deaths were a consequence of either multiorgan dysfunction related to cardiogenic shock, overwhelming sepsis, or refractory ventricular arrhythmias. Only two studies assessed costs. Chihrin et al. found that, in the first 18 hours of use, the costs of TPPM placement were higher due to the price of the active fixation lead.^34^ The price of the pacemaker generator was not included, as it is reusable. After this period, they concluded a TPPM would save $456 per 24-hour interval in comparison with passive fixation leads. Lever et al. also reported reduced costs with TPPM placement.^39^ Obviating the need to use a bed in the cardiac care unit likely reduces costs related to the provision of an advanced level of care.

All studies used VVI pacing except for one that used VDD,^40^ and eight described the use of atrial pacing. Pang et al. reported on two patients who were paced in VVI mode and who became hypotensive due to atrioventricular dyssynchrony.^41^ After placement of an atrial lead, they improved clinically. Orsbourn et al. also reported on the use of dual-chamber pacing in seven of the 23 patients they studied.^42^ Lepillier et al. followed eight patients with complete heart block and heart failure who had temporary dual-chamber pacemakers placed and observed an improvement in heart failure symptoms and brain natriuretic peptide levels.^40^ Level of activity was reported in 10 studies **(Table 4)**. Some patients had to remain in bed despite TPPM placement because of other comorbidities.^31,35,43^

In two studies by De Cock et al., ambulation was reported as occurring in 75% and 73%.^29,30^ Spontaneous loss of capture was not documented. One patient removed his pacing lead secondary to delirium. Garcia et al. prospectively assessed 47 patients who had received a femoral TPPM^31^ and classified them into the categories of high, moderate, and low mobility. Only three out of the 12 patients in the low-mobility group had a DVT, while such was not documented at all in those with medium or high mobility. They compared their findings with those from an older study with an incidence of 25% to 39% of asymptomatic DVT achieved when using passive fixation leads.^44^ De Cock et al. also reported that only one out of 42 patients developed DVT.^29^ All of these patients were being anticoagulated with intravenous heparin, which likely confounded the outcome.

Two of the reviewed studies had a group with passive fixation leads for comparison with the TPPM group.^30,35^ Braun et al. in 2006 compared 23 patients treated with TPPM placement and 26 treated with a passive fixation lead. Infection was not reported in either group. There were 24 “loss-of-capture” events in the passive fixation group versus one in the active fixation group (p < 0.01). Three patients in the first group required resuscitation on more than one occasion, which prompted pacing with a TPPM.

## Discussion

Thanks to a screw-in mechanism, the active fixation lead provides greater stability and reliable pacing.^9,38,45^ Intermittent loss of capture during temporary pacing is a relatively common cause of intensive care unit (ICU) emergencies in part because prolonged pacing can suppress ventricular escape and precipitate asystole if loss of capture occurs.^46^ The added results of our review show a 1.7% dislodgement rate for TPPM. This benefit was noticeable even when TPPM was used for months.^38,45^ The value of this finding remains in patients who might require temporary pacing for long periods of time.^32,33^

Passive leads are often used in patients who are hemodynamically unstable and who cannot be transported to a procedure room. The parameters used in assessing proper placement are length of lead inserted, telemetry monitoring that confirms ventricular capture, and chest X-ray.^37^ Screw-in leads ideally require transferring the patient to the catheterization laboratory for placement under fluoroscopy to ensure that the screw is deployed in the proper position. The dislodgement rate when using passive fixation leads has been reported at 17% with femoral leads after 4.8 days of follow-up in a series of 100 patients,^47^ while other studies have suggested it to be between 10% and 30%.^48,49^

Pacing thresholds when using TPPM have been reported to be less than 1 V in most studies.^26,38,42,43,50^ Similar to the placement of permanent pacemakers or passive fixation leads, a low capture threshold is one of the parameters used to determine proper placement of the pacing electrode. It is recommended that pacing and sensing be programmed in a bipolar fashion, since the pacemaker generator is externalized.^50^

The use of temporary pacing allows for the safe removal of an infected device, particularly in patients who are pacemaker-dependent.^51^ After the infected device has been explanted, there needs to be a delay for implanting a new device starting from the first set of negative blood cultures, and this period of time is subjected to the presence of valvular endocarditis and extracardiac bacterial seeding.^52^ Although small studies have shown good outcomes with the removal of an infected device and simultaneous placement of a new one, the availability of reliable temporary pacing using TPPMs does not justify managing patients in such a manner. In one study, Nandyala and Parsonet followed 68 patients with CIEDs and did not use TPPMs prior to extraction, instead implanting a new device at the contralateral site simultaneously. After a follow-up of more than one year, no recurrent infections were found.^53^ Another retrospective review of 15 patients with same-day device implantation after lead extraction showed no recurrence of infection after a median follow-up of 44 months.^54^ Simultaneous lead extraction and implantation of epicardial leads has also been reported in conjunction with good long-term outcomes,^55^ with an overall complication rate similar to that of the transvenous route.

Concurrent infection of the temporary pacemaker can occur and, here, TPPMs appear to become infected less often than passive fixation leads. Most of the TPPM infections that we found were reported in research by De Cock et al., where transvenous femoral access was used routinely.^29,30^ It has been well-described that there is an increased risk of infection from femoral venous lines, with the lowest being subclavian.^56^ Among the reasons for why TPPMs may have a lower incidence of infection, one could consider the reduced manipulation of the lead, since loss of capture is infrequent and the entry site through the skin is smaller because a sheath does not have to be left in place, therefore minimizing bacteria seeding into the bloodstream.^36,50^ The presence of comorbidities and the duration of pacing were similar when active and passive fixation lead cases were compared.^30,35^

In one center, all TPPMs were placed with tunneled leads, with no report of secondary infections.^42^ At this time, due to the low rate of infection associated with TPPMs, it is difficult to recommend the routine use of tunneled leads. Such may be considered in patients who are expected to use TPPMs for a very long period of time or who have other risk factors.

TPPMs are routinely placed contralaterally to the site where the permanent pacemaker is wanted. The right internal jugular vein is often approached in order to protect the subclavian veins that are generally used for permanent pacing.^24^ Pneumothorax risk is low with internal jugular access guided by ultrasound, while the same risk during subclavian access can be minimized with ultrasound and fluoroscopic guidance.^57^

It is still debatable as to whether the same site where the infected device was can be used for placement of a TPPM.^56^ Some authors have explored placement of a temporary pacemaker through the same site where the infected pacemaker was, with the advantage of the new permanent device being located far from where the prior infection was found.^28,39,58^ A potential disadvantage of this approach could be an increased risk for infection of the TPPM itself.

The procedure to place a TPPM is similar to that of a permanent pacemaker, with the exception of that a subcutaneous pocket is not needed.^6^ Preparation and aseptic techniques are similar to those of placing a central venous catheter.^59^ The anatomical landmark used when approaching the internal jugular vein is the angle between the two heads of the sternocleidomastoid muscle. Ultrasound will show the internal jugular vein and the common carotid artery, with the former being much more compressible. With ultrasound, we also can see the needle in real time as it advances through tissue. Once access is obtained, a J-shaped guidewire is advanced and a peelable sheath is threaded through it. Under fluoroscopic guidance, a pacemaker lead with a preformed stylet inside is advanced into the right ventricle and the screw is deployed either in the apex or the septum. Testing is done to ensure appropriate sensing, impedance, and capture thresholds. Once done, the sheath is peeled away and the lead is secured to the skin through the suture sleeve. The proximal end of the lead is inserted in the can and screwed, and the latter is finally attached to the patient’s skin with sutures and/or adhesives.

Some studies have addressed the use of temporal dual-chamber pacing.^40–42^ This seems to be of the utmost importance in the setting of critical illness and known heart disease, where maintaining atrioventricular synchrony and optimal cardiac output becomes significant. Right ventricular pacing can cause atrioventricular dissociation leading to pacemaker syndrome as well as interventricular dyssynchrony with reduction of the left ventricular systolic function.^24,38^ Dual pacing can also be achieved with a balloon-tipped single lead that includes noncontact atrial dipoles and which can perform overlapping biphasic impulse stimulation.^60^ A caveat to routinely placing two leads instead of one is the potential for an increased risk of infection and thrombosis. It would be prudent to pace both the atrium and ventricle only when a significant hemodynamic benefit is expected.

Patients can be discharged from the hospital while still using a TPPM^38,45,56^ and ambulation can often be resumed quickly.^30,36,39,45^ This is not so in the case of passive fixation leads, which require a patient to be on bed rest and telemetry for 24 hours per day. The disadvantages of remaining on bed rest for long periods of time are well-described and include a risk for DVT, deconditioning, atelectasis, and increased hospital stay, among others. This becomes more important in patients who require prolonged temporary pacing such as those with CIED-related endocarditis. Ambulation in these patients is also promoted by the smaller size of the resterilized generator.^61^

Loss of capture can still occur with active fixation leads, such as when a patient moves abruptly or during a lead extraction procedure.^24^ Unintended dislodgement of the temporary lead could be prevented by positioning it at a certain distance from the leads to be extracted.^51^

Most of the deaths documented were related to patient comorbidities. As an example, one study revealed that death was more frequent in patients who had a TPPM placed for an indication that was one other than infection of a CIED.^24^ This is likely the case because most CIED infections are limited to the pocket site. In another example, Noble et al. reported the use of TPPM in 20 patients who had undergone transaortic valve replacement, a population that is expected to have a better outcome than those hospitalized in the ICU.^62^ Here, there were only two deaths that occurred and none of these were secondary to the device itself. On the other hand, Dawood et al. reported a 29.6% mortality rate from etiologies that included ventricular fibrillation, respiratory failure, non-ST-segment-elevation myocardial infarction, abdominal aortic aneurysm rupture, stroke, and subdural hematoma.^63^

Despite the fact that TPPMs were used for prolonged periods of time, such still was superior in terms of overall complication rates to conventional temporary pacemakers,^24,36^ which have been reported to have rates as high as 30%.^9,14,64^

The duration of hospital stay was rather prolonged with TPPM usage, likely from the underlying comorbidities.^24^ If there was no other indication to continue being in the hospital, patients with TPPMs were usually able to leave for home or a nursing facility. This was not possible in those with passive fixation leads, since the indication for pacing had to be reversed to remove the temporary pacemaker or the patient need undergo placement of a permanent device. The shortest hospital stay was reported by Noble et al. (mean: 11.3 ± 4.7 days), while the longest was noted by Kornberger et al. (mean: 30.7 ± 23.8 days).^37,62^

Few studies reported on the use of TPPMs that also were defibrillators. At present, it is difficult to justify this approach when wearable cardioverter-defibrillators are available, although it is common to learn that patients do not wear them consistently because of discomfort. Cooper et al. reported the case of a patient with an infected device who had multiple episodes of sustained ventricular tachycardia.^43^ The external device used was a pacemaker and a defibrillator that allowed for the termination of these episodes with antitachycardia pacing with the avoidance of defibrillation.

Costs may be significantly reduced using active fixation leads. Only one publication at this time appears to have specifically addressed this question.^36^ The reduction in costs was mainly determined by the reduced length of stay in the cardiac care unit and by obviating the use of telemetry. One problem with this study, however, is that it involved mainly patients with sleep apnea who volunteered to have a TPPM implanted and who would not have any other indication to stay in the ICU. If more ill patients were included, then a clear cost benefit may have not been as evident. Lever et al. also concluded that TPPM placement is associated with less costs.^39^

The duration of TPPM use was variable, with some cases being as long as months and with a good safety margin. Certainly, the reliability of the active fixation mechanism allows for application for such extended periods. This can be justified in patients who remain critically ill who require a permanent pacemaker and who are at a high risk of complications if transported to a procedure room. In some uncommon situations, patients experienced a recovery of their conduction abnormalities after a lengthy hospital stay.^36,46,49^ It may be wise to use a TPPM for as brief a period as possible in patients who have prosthetic material in their bodies due to the potential of bacterial seeding. These patients include those who undergo transcatheter aortic valve replacement, a population in which TPPMs are used frequently.^65^

## Conclusions

TPPMs constitute a safe modality for temporary pacing. The associated fixation mechanism and fairly easy placement make this type of device a superior option over conventional temporary pacing. We recommend it should be used as first-line and that passive fixation leads be limited to use in patients who are not stable enough to be transferred to a room with fluoroscopy.

However, it is important to note that most studies considered herein involved a small sample size and were single-center. Many did not report the time of follow-up. Designs were heterogeneous, hindering their comparison. Follow-up was reported in only nine studies. There was a comparison group with passive fixation leads in only two studies; thus, most authors compared their data to historical references. With the information available, we were unable to separate the critically ill from the noncritically ill individuals so as to establish the mortality rate for each.

Additionally, Chihrin et al. were the only authors to compare costs with nonactive fixation leads. Some studies assessed ambulation after TPPM placement, with four studies quantifying the number of patients who ambulated. Two studies assessed the presence of DVT with a large bias, since these used femoral venous access, and the majority of patients were anticoagulated with heparin. Few studies explored the use of dual-chamber and atrial pacing, knowing the potential hemodynamic benefits of maintaining atrioventricular synchrony.

## Figures and Tables

**Table 1:** Indications for TPPM Use

Pacemaker or ICD infection
Sick sinus syndrome
Complete heart block
Medicine washout
Transcatheter aortic valve replacement
Ventricular tachycardia
Bradyarrhythmias associated with critical illness
Pacemaker syndrome
New or alternating bundle branch block
Guillain–Barré syndrome

ICD: implantable cardioverter-defibrillator.

**Table 2:** Number of Patients, Follow-up Period, and Duration of Temporary Pacing per Study

Study	Number of Patients	Follow-up Period	Duration of Temporary Pacing
Amraoui et al. 2015^24^	80	1 year	4–14 days
Martin et al. 1999^26^	21	Not reported	Mean: 12.5 (1–32 days)
Pecha et al. 2013^27^	17	Mean: 21.2 (12–36) months	Mean: 12.7 (6–24) days
Zei et al. 2006^28^	62	Not reported	Median: 7.5 days
De Cock et al. 2003^29^	72	Not reported	Control group: 5.84 ± 2.4 days Validation group: 5.94 ± 2.6 days
De Cock et al. 2003^30^	42	Not reported	Mean: 5.96 ± 2.6 days
Garcia et al. 2010^31^	47	Not reported	Mean: 5.9 (2–25 days)
Pinto et al. 2003^32^	4	Not reported	Median: 19.5 (5–38) days
Rastan et al. 2005^34^	10	Not reported	13.5 ± 10.5 days
Braun et al. 2006^35^	49	Reference median: 12 (1–29) days External median: 14 (3–45) days	Reference group: 1–19 days External group: 2–18 days
Chihrin et al. 2006^36^	20	1 month	Median: 2 (2–83) days
Kornberger et al. 2013^37^	59	12 months (only CIED group)	Mean: 14.6 ± 8.1 days
Kawata et al. 2013^38^	23	Mean: 7.1 ± 5.9 months	Median: 18 (19.4 ± 11.8) days
Lever et al. 2003^39^	20	Not reported	Median: 28 (9–81) days
Lepillier et al. 2012^40^	8	Mean: 15.8 ± 5.3 months	8 ± 2.5 days
Pang et al. 2012^41^	3	Not reported	Mean: 9.3 days
Orsbourn et al. 2008^42^	23	Not reported	Median: 16 (2–71) days
Cooper et al. 2011^43^	1	Not reported	5 weeks
Lang et al. 2005^50^	1	Not reported	120 days
Maciag et al. 2015^51^	34	Not reported	Mean: 14.5 (4–26) days
Arias et al. 2012^61^	1	Not reported	21 days
Noble et al. 2011^62^	20	Not reported	Mean: 5.6 ± 1.9 days
Dawood et al. 2016^63^	152	At least 6 months for the mortality rate	Not reported (time to PPM implant was reported as a mean of 9.7 days and a median of 21 days)
Cuisset et al. 2011^65^	1	5 days	5 days

**Table 3:** Complications Secondary to TPPM Implantation and Use

Study	Infections	Loss of Capture	Deaths	Total Number of Complications*
Amraoui et al. 2015^24^	0	0	4 (not related to TPPM use)	2
Martin et al. 1999^26^	1	0	2 (not related to TPPM use)	1
Pecha et al. 2013^27^	0	0	0	0
Zei et al. 2006^28^	0	0	11 (not arrhythmia-related)	0
De Cock et al. 2003^29^	5	2	0	11
De Cock et al. 2003^30^	Control group: 4 (11%) Validation group: 6 (16%)	Control group: 12 (36.4%) Validation group: 2 (5.5%)	0	Control group: 21 Validation group: 11 (p < 0.01)
Garcia et al. 2010^31^	2	0	3	8**
Pinto et al. 2003^32^	0	0	0	0
Rastan et al. 2005^34^	0	0	0	0
Braun et al. 2006^35^	Reference group: 0 External group: 0	Reference group: 24 External group: 1	Reference group: 4 External group: 3	Reference group: 28 External group: 6
Chihrin et al. 2006^36^	0	1	0	1
Kornberger et al. 2013^37^	0	2	5 (not related to TPPM use)	6
Kawata et al. 2013^38^	1	0	1 (not related to TPPM use)	1
Lever et al. 2003^39^	2	0	1 (not related to TPPM use)	2
Lepillier et al. 2012^40^	0	0	0	0
Pang et al. 2012^41^	0	0	0	0
Orsbourn et al. 2008^42^	0	0	4 (not related to TPPM use)	0
Cooper et al. 2011^43^	0	0	0	0
Lang et al. 2005^50^	1	0	0	1
Maciag et al. 2015^51^	0	Not reported	3	0
Arias et al. 2012^61^	0	0	0	0
Noble et al. 2012^62^	0	Not reported	2 (not clear if TPPM-related)	0
Dawood et al. 2016^63^	0	0	45 (not related to TPPM use)	1
Cuisset et al. 2011^65^	0	0	0	0
Total***	18	8	84 (6 were TPPM-related)	49

TPPM: temporary permanent pacemaker.

*Total complications do not include deaths.

**Three cases of increased threshold included that were treated by increasing output.

***Total values exclude patients in the reference and control groups.

**Table 4:** Ambulation with TPPM

Study	Ambulation Details
Zei et al. 2006^28^	• Immediate ambulation encouraged
De Cock et al. 2003^29^	• Control group: Bed rest • Validation group: 75% within in one hour
De Cock et al. 2003^30^	• 73% of patients ambulated
Garcia et al. 2010^31^	• High mobility: 29 patients • Minimum mobility: 6 patients • Bed rest: 12 patients
Braun et al. 2006^35^	• All patients were confined to bed rest
Kornberger et al. 2013^37^	• Only mentioned that patients with TPPMs ambulated
Lever et al. 2003^39^	• Only mentioned that patients with TPPMs ambulated
Orsbourn et al. 2008^42^	• Only mentioned that patients with TPPMs ambulated
Cooper et al. 2011^43^	• Patient intubated
Arias et al. 2012^61^	• Patients ambulated at 24 hours

TPPM: temporary permanent pacemaker.
